# Dystrophin Gene Mutation Location and the Risk of Cognitive Impairment in Duchenne Muscular Dystrophy

**DOI:** 10.1371/journal.pone.0008803

**Published:** 2010-01-20

**Authors:** Peter J. Taylor, Grant A. Betts, Sarah Maroulis, Christian Gilissen, Robyn L. Pedersen, David R. Mowat, Heather M. Johnston, Michael F. Buckley

**Affiliations:** 1 Molecular and Cytogenetics Unit, Department of Haematology and Genetics, Prince of Wales Hospital, Randwick, Sydney, New South Wales, Australia; 2 Sydney Neuromuscular Centre, Sydney Children's Hospital, Randwick, Sydney, New South Wales, Australia; 3 Department of Human Genetics, Radboud University Nijmegen Medical Centre, Nijmegen, The Netherlands; 4 The School of Women's and Children's Health, University of New South Wales, Sydney, New South Wales, Australia; University Hospital Vall d'Hebron, Spain

## Abstract

**Background:**

A significant component of the variation in cognitive disability that is observed in Duchenne muscular dystrophy (DMD) is known to be under genetic regulation. In this study we report correlations between standardised measures of intelligence and mutational class, mutation size, mutation location and the involvement of dystrophin isoforms.

**Methods and Results:**

Sixty two male subjects were recruited as part of a study of the cognitive spectrum in boys with DMD conducted at the Sydney Children's Hospital (SCH). All 62 children received neuropsychological testing from a single clinical psychologist and had a defined dystrophin gene (*DMD*) mutation; including *DMD* gene deletions, duplications and DNA point mutations. Full Scale Intelligence Quotients (FSIQ) in unrelated subjects with the same mutation were found to be highly correlated (*r* = 0.83, *p* = 0.0008), in contrast to results in previous publications. In 58 cases (94%) it was possible to definitively assign a mutation as affecting one or more dystrophin isoforms. A strong association between the risk of cognitive disability and the involvement of groups of *DMD* isoforms was found. In particular, improvements in the correlation of FSIQ with mutation location were identified when a new classification system for mutations affecting the Dp140 isoform was implemented.

**Significance:**

These data represent one of the largest studies of FSIQ and mutational data in DMD patients and is among the first to report on a DMD cohort which has had both comprehensive mutational analysis and FSIQ testing through a single referral centre. The correlation between FSIQ results with the location of the dystrophin gene mutation suggests that the risk of cognitive deficit is a result of the cumulative loss of central nervous system (CNS) expressed dystrophin isoforms, and that correct classification of isoform involvement results in improved estimates of risk.

## Introduction

Duchenne muscular dystrophy (DMD) is a clinically heterogeneous disorder of at least 4 clinical subphenotypes characterised by differences in the severity of muscle and brain dysfunction (Online Mendelian Inheritance in Man (OMIM) 310200) [1]. Duchenne de Boulogne first noted the presence of cognitive deficits in DMD in his initial description of the disorder [2], an observation which has been confirmed in many subsequent studies [3]–[5]. The consistent finding in the DMD neuropsychological profile is a reduction in mean FSIQ by approximately 1 standard deviation with respect to the population mean; with a range of severity from borderline neuropsychological deficits to severe intellectual disability. The frequency of FSIQ<70 has been estimated to be in the range of 19–35% of DMD cases, with 3% of patients having moderate-severe intellectual disability (FSIQ<50) [4]–[6]. Several studies have compared performance intelligence quotients (PIQ) with verbal intelligence quotients (VIQ). Most studies are in agreement that VIQ is more affected than PIQ and that the difference of their means is about 5–8 points [7]–[10], although other authors have maintained that the deficits are global in nature [11], [12]. Several studies have performed more detailed analyses of the specific areas of verbal intelligence that are most affected and have shown that the impairment of verbal ability appears to be caused by a defect in verbal working memory for patterns, numbers and verbal labels [13]–[16].

Prior to 1960 the cause of cognitive disability in DMD patients was primarily attributed to functional disabilities or social environment [17]. The initial evidence of a genetic contribution to the cognitive defects was provided by the observation of a greater concordance for IQ among affected brothers. In their study of 84 siblings with DMD, Ogasawara *et al* reported a correlation of 0.80 [18], which was significantly higher than the median correlation of 0.38 (range 0.0–0.55) identified in a meta-analysis of 12 studies of IQ in unaffected male siblings raised together [19]. Comparisons between DMD and spinal muscular atrophy (SMA) patients showed that the DMD group had consistently lower IQ scores and memory skills. Subsequently, Billard and colleagues confirmed these findings and further established that DMD boys performed poorly in reading tasks and in specific memory functions when compared to age matched SMA patients [13], [20]. A significant difference in the frequency of cognitive impairment is also present between the allelic disorders Duchenne and Becker muscular dystrophy [21]. Despite considerable evidence that the deficit in intellectual function in DMD has a significant genetic component a simple relationship between the degree of cognitive impairment and the severity of muscle weakness has not been identified, suggesting that these interrelated phenotypes are under a degree of tissue specific control.

**Figure 1:**
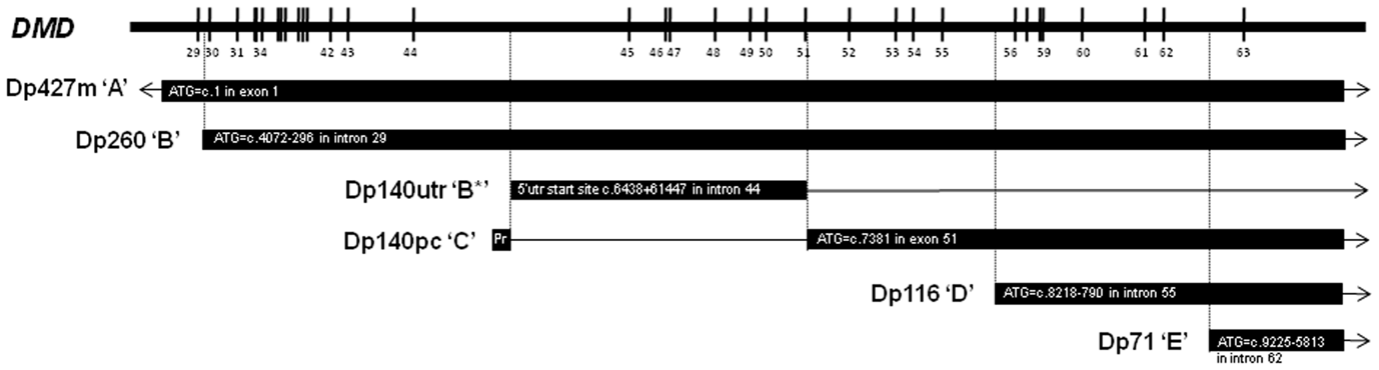
Genomic organisation of alternate dystrophin isoforms. The relationship between the abbreviated nomenclature used for the isoforms affected by a *DMD* mutation and the structure of the Dp427m, Dp260, Dp140 and Dp116 isoforms of the dystrophin gene. The black vertical lines represent the coding exons of the dystrophin gene with exon numbers given below. The positions of the initiator Methionine (ATG), untranslated region start site (utr) and promoter (Pr) are depicted. The figure demonstrates that exons 45–50 together with the 5′ region of exon 51 lie within the 1.041Kbp 5′UTR of Dp140 as well as within the coding regions of Dp260 and Dp427. The numbering used is with respect to intron/exon structure of Dp427m NM_004006.2 and the Human Genome reference sequence of Ensembl build 52 (Dec 2008) implemented in Alamut version 1.5.

DMD is the clinical manifestation of diverse mutational events within the *DMD* gene that result in absence of functional dystrophin protein. The *DMD* locus produces at least 7 major dystrophin isoforms from 7 recognised promoters which exhibit developmental, regional and cell-type specificity within the central nervous system. Three full-length isoforms are derived from unique upstream promoter/first exon sequences Dp427c (also referred to by some authors as Dp427b), Dp427m, and Dp427p. At least four shorter mRNA products Dp260, Dp140, Dp116 and Dp71 are transcribed from more distal promoters located within downstream introns of *DMD* (Figure 1) [22]. Among these, Dp71 and Dp140 are particularly abundant in foetal brain, which has led to the suggestion that they may be of particular significance to the cognitive defects in DMD [23].

Evidence of a link between mutation location within *DMD* and cognitive deficit was based initially on the observation that deletions of exon 52 were associated with cognitive impairment [24]. Subsequent studies by Bushby and colleagues reported that rather than the cognitive impairment being specifically associated with deletions involving exon 52, deletions localised in the second half of the gene were more frequently associated with lower IQ than those in the first half of the gene, but no specific genotype/phenotype relationship was identified [25], [26]. A relationship between the intellectual impairment and altered expression of the C-terminal brain-expressed dystrophin isoforms was suggested by several case reports [27]. The loss of two of the shorter isoforms of dystrophin, Dp140 and Dp71, has been reported to have the greatest impact on IQ in DMD [28], whereas there has been no association reported regarding loss of the Dp260 or Dp116 isoforms. Mutations that affect Dp260 expression have however been associated with the liability of ophthalmic involvement [29]. Mutations of the promoter of the Dp140 isoform have been implicated in the risk of cognitive disability [30], [31].

There is now substantial evidence that despite their rarity all patients with mutations involving the Dp71 isoform have severe intellectual disability [27], [32]–[34] and it has been hypothesised that as more distal mutations have the potential to affect the expression of increasing numbers of dystrophin isoforms the severity and frequency of intellectual disability is related to the effect of cumulative loss of functional distal isoforms [33].

Over the past 15 years only a minority of the published studies which have attempted to correlate mutational and IQ data have reported mutations across the full mutation spectrum seen in the DMD gene [1], [26], [32]–[36]. Here we report correlations between standardised measures of intelligence and mutational class, mutation size, mutation location and the cumulative loss of dystrophin isoforms, focusing in particular on the risk of cognitive disability for mutations that involve the dystrophin isoform Dp140.

## Results

Results for 62 male DMD subjects are provided in Table 1, including data relating to the identified mutation, the exons affected by the mutation, the location of a predicted premature termination codon, the isoform(s) affected as inferred from mutation location, and results of the neuropsychological assessments. As discussed in detail below (see Subjects and Methods) the nomenclature system used to relate mutation location to *DMD* isoform is depicted in Figure 1. In particular the designations B* (Table 1, Figure 1) and Dp140utr (in the text) are used for mutations located in the extended 5′UTR of the Dp140 isoform and which leave the Dp140 promoter intact, whereas the designations C (Table 1, Figure 1) and Dp140pc (text) areused to indicate mutations that affect the promoter and/or coding region of the Dp140 isoform.

**Table 1:** 

*DMD* Mutation Position (NM_004006.2)	Predicted Effect of Mutation	In-frame/out-of-frame	Pathogenic Event	Isoform(s)	Age	FSIQ	VIQ	PIQ	VIQ -PIQ
c.32-?_93+?dup	Duplication of exon 2	Out of Frame	PTC 45bp into duplicated exon 2	A	6.5	80	74	90	−16
c.32-?_93+?dup	Duplication of exon 2	Out of Frame	PTC 45bp into duplicated exon 2	A	9.1	130	131	123	8
c.94-?_264+?dup	Duplication of exons 3–4	In Frame	Partial duplication of actin binding domain [37]	A	10.1	113	N/A	N/A	N/A
c.94-?_831+?dup	Duplication of exons 3–8	In Frame	Partial duplication of actin binding domain [37]	A	6.0	98	86	111	−25
c.94-?_3603+?del	Deletion of exons 3–26	In Frame	Partial deletion of actin binding and repeat spectrin domains [37]	A	11.1	104	113	93	20
c.433C>T	Nonsense mutation [p.Arg145X]	Point mutation	PTC at c.435 in exon 6	A	11.3	109	102	115	−13
c.433C>T	Nonsense mutation [p.Arg145X]	Point mutation	PTC at c.435 in exon 6	A	11.3	131	121	139	−18
c.265-?_649+?del	Deletions of exons 5–7	Out of Frame	PTC at c.688 in exon 8	A	9.4	99	98	102	−4
c.[94-?_1331+?del+ 2623-?_4233+?dup]	Deletion of exons 3–11, *and* duplication of exons 21–30	Out of Frame	PTC at c.1340 in exon 12	A	14.8	79	83	79	4
c.961-?_1331+?del	Deletion of exons 10–11	Out of Frame	PTC at c.1340 in exon 12	A	7.7	51	52	58	−6
c.650-?_1602+?del	Deletion of exons 8–13	Out of Frame	PTC at c.1634 in exon 14	A	7.1	88	84	95	−11
c.961-?_1602+?del	Deletion of exons 10–13	In Frame	Partial deletion of spectrin repeat domain [37]	A	7.6	103	99	107	−8
c.1699G>T	Nonsense mutation [p.Glu567X]	Point mutation	PTC at c.1701 in exon 14	A	11.2	84	69	104	−35
c.94-?_2168+?del	Deletion of exons 3–17	Out of Frame	PTC at c.2180 in exon 18	A	11.3	71	80	68	12
c.2169-?_2292+?del	Deletion of exon 18	Out of Frame	PTC at c.2299 in exon 19	A	14.2	105	97	N/A	N/A
c.650-?_2292+?del	Deletion of exons 8–18	Out of Frame	PTC at c.2315 in exon 19	A	8.0	104	91	119	−28
c.2665C>T	Nonsense mutation [p.Arg889X]	Point mutation	PTC at c.2667 in exon 21	A	7.2	104	100	107	−7
c.2665C>T	Nonsense mutation [p.Arg889X]	Point mutation	PTC at c.2667 in exon 21	A	7.3	83	73	96	−23
c.2293-?_2803+?del	Deletion of exons 19–21	Out of Frame	PTC at c.2845 in exon 22	A	11.9	73	71	79	−8
c.2974C>T	Nonsense mutation [p.Gln992X]	Point mutation	PTC at c.2976 in exon 23	A	4.3	62	53	77	−24
c.2991C>G	Nonsense mutation [p.Tyr997X]	Point mutation	PTC at c.2991 in exon 23	A	11.3	75	84	69	15
c.3086G>A	Nonsense mutation [p.Trp1029X]	Point mutation	PTC at c.3087 in exon 23	A	11.2	101	101	100	1
c.5449-1G>C	Splicing mutation at exon 39	Splice mutation	incorrectly spliced mRNAs	AB	7.5	88	79	100	−21
c.1332-?_5922+?dup	Duplication of exons 12–41	Out of Frame	PTC 9bp into duplicated exon 12	AB	7.4	72	66	82	−16
c.650-?_5922+?del	Deletion of exons 8–41	Out of Frame	PTC at c.5936 in exon 42	AB	11.2	84	88	83	5
c.94-?_6290+?del	Deletion of exons 3–43	Out of Frame	PTC at c.6299 in exon 44	AB	7.8	78	82	79	3
c.6291-?_6438+?del	Deletion of exon 44 (Dp140pr/ex1 intact)	Out of Frame	PTC at c.6487 in exon 45	ABB*	7.5	70	73	72	1
c.6439-?_6614+?del	Deletion of exon 45 (Dp140pr/ex1 intact)	Out of Frame	PTC at c.6665 in exon 46	ABB*	11.3	123	124	117	7
c.6439-?_6614+?del	Deletion of exon 45 (Dp140pr/ex1 intact)	Out of Frame	PTC at c.6665 in exon 46	ABB*	9.8	103	104	102	2
c.6439-?_7309+?del	Deletion of exons 45–50 (Dp140pr/ex1 intact)	Out of Frame	PTC at c.7226 in exon 50	ABB*	11.0	92	91	95	−4
c.6439-?_7309+?del	Deletion of exons 45–50 (Dp140pr/ex1 intact)	Out of Frame	PTC at c.7226 in exon 50	ABB*	5.2	84	83	90	−7
c.6615-?_7200+?del	Deletion of exons 46–49	Out of Frame	PTC at c.7210 in exon 50	ABB*	11.1	65	63	73	−10
c.6615-?_7200+?del	Deletion of exons 46–49	Out of Frame	PTC at c.7210 in exon 50	ABB*	7.2	93	88	100	−12
c.6913-?_7309+?del	Deletion of exons 48–50	Out of Frame	PTC at c.7336 in exon 51	ABB*	7.2	84	82	89	−7
c.6913-?_7309+?del	Deletion of exons 48–50	Out of Frame	PTC at c.7336 in exon 51	ABB*	7.2	118	105	130	−25
c.6913-?_7309+?del	Deletion of exons 48–50	Out of Frame	PTC at c.7336 in exon 51	ABB*	7.5	97	93	102	−9
c.7099-?_7309+?del	Deletion of exons 49–50	Out of Frame	PTC at c.7336 in exon 51	ABB*	10.2	69	64	78	−14
c.7099-?_7309+?del	Deletion of exons 49–50	Out of Frame	PTC at c.7336 in exon 51	ABB*	11.1	73	69	82	−13
c.7099-?_7309+?del	Deletion of exons 49–50	Out of Frame	PTC at c.7336 in exon 51	ABB*	9.3	79	83	79	4
c.7099-?_7309+?del	Deletion of exons 49–50	Out of Frame	PTC at c.7336 in exon 51	ABB*	9.3	84	83	89	−6
c.6291-?_6438+?del	Deletion of exon 44 (Dp140pr/ex1 deleted)	Out of Frame	PTC at c.6487 in exon 45	ABC	9.1	75	73	81	−8
c.6291-?_7098+?del	Deletion of exons 44–48	Out of Frame	PTC at c.7111 in exon 49	ABC	5.9	86	93	80	13
c.6615-?_7542+?del	Deletion of exons 46–51	Out of Frame	PTC at c.7612 in exon 52	ABC	7.3	87	79	100	−21
c.7310-?_7542+?del	Deletion of exon 51	Out of Frame	PTC at c.7640 in exon 52	ABC	14.4	62	76	N/A	N/A
c.7099-?_7660+?del	Deletion of exons 49–52	Out of Frame	PTC at c.7726 in exon 53	ABC	4.8	96	105	93	12
c.7543-?_7660+?del	Deletion of exon 52	Out of Frame	PTC at c.7726 in exon 53	ABC	14.6	71	75	71	4
c.7689delA	Frameshift [p.Val2564TyrfsX12]	PTC	PTC at c.7726 in exon 53	ABC	6.0	71	81	65	16
c.7689delA	Frameshift [p.Val2564TyrfsX12]	PTC	PTC at c.7726 in exon 53	ABC	7.2	90	81	102	−21
c.7661-2A>C	Splicing mutation at exon 53	Splice mutation	incorrectly spliced mRNA	ABC	11.5	52	N/A	64	N/A
c.7873-?_8027+?del	Deletion of exon 54	Out of Frame	PTC at c.8036 in exon 55	ABC	11.0	84	73	99	−26
c.7873-?_8027+?del	Deletion of exon 54	Out of Frame	PTC at c.8036 in exon 55	ABC	14.5	71	81	65	16
c.8217+5G>A	Splicing mutation at exon 55	Splice mutation	incorrectly spliced mRNA	ABCD	14.1	69	64	78	−14
c.8259delC	Frameshift [p.His2753GlnfsX11]	PTC	PTC at c.8290 in exon 56	ABCD	5.0	87	78	102	−24
c.8608C>T	Nonsense [p.Arg2870X]	PTC	PTC at c.8610 in exon 58	ABCD	4.8	61	63	67	−4
c.9224+1G>A	Splice mutation at exon 62	Splice mutation	incorrectly spliced mRNA	ABCD	14.0	79	82	80	2
c.9204_9207delCAAA	Frameshift [p.Asn3068LysfsX20]	PTC	PTC at c.9265 in exon 63	ABCD	10.0	47	56	46	10
c.9085-?_9286+?del	Deletion of exons 61–63	Out of Frame	PTC at c.9295 in exon 64	ABCDE	9.5	42	46	47	−1
c.10609delT	Frameshift [p.Ser3537ProfsX9]	Point mutation	PTC at c.10636 in exon 75	ABCDE	13.7	55	50	68	−18
c.6439-?_6614+?dup	Duplication of exon 45	Out of Frame	Isoforms not assigned		7.2	80	73	91	−18
c.6439-?_7660+?dup	Duplication of exons 45–52	Out of Frame	Isoforms not assigned		11.1	46	N/A	N/A	N/A
c.6439-?_7660+?dup	Duplication of exons 45–52	Out of Frame	Isoforms not assigned		11.1	50	N/A	N/A	N/A
c.6439-?_7660+?dup	Duplication of exons 45–52	Out of Frame	Isoforms not assigned		14.8	50	N/A	N/A	N/A

Del = exon deletion mutation, Dup = exon duplication mutation, PTC = Premature Termination Codon. Dp140pr/ex1 = Dp140 promoter and exon 1.

Dystrophin isoforms: A = Dp427m; B = Dp260; B* = Dp140utr; C = Dp140pc; D = Dp116; E = Dp71. N/A = not available.

All subjects were ascertained based on a clinical diagnosis of DMD supported by the identification of a mutation in the *DMD* gene. Mutation analysis of the *DMD* gene identified 58 out-of-frame and 4 in-frame mutations at the genomic level (Table 1) consistent with the general expectation that mutations associated with the DMD phenotype produce premature protein truncation or instability of the dystrophin protein. It is notable that the four in-frame mutations identified in this cohort have been previously reported as being associated with DMD and are clustered at the 5′ end of the gene, either within or adjacent to the actin binding domain of the protein [37]. It is inferred that these disrupt critical domain functions or destabilise the dystrophin protein.

### Descriptive IQ Statistics in the SCH DMD Cohort

Significant differences were observed within the SCH cohort compared with standardised normative values for FSIQ, PIQ and VIQ (p<0.0001) as assessed by one-sample *t*-test analysis, consistent with previous reports [4]. The frequency of intellectual disability (FSIQ<70) in the SCH study group was 24.2% (15/62). The reported discordance between VIQ and PIQ was also identified in this study and had a mean value of −6.7 (*N* = 55, SD = 13.2). This is significantly different from the standardised mean value of zero (t = 3.768 df = 54, p<0.0005), indicating that verbal intelligence is more affected than performance intelligence in children with DMD, consistent with previous published studies [8], [9], [12]. Five patients (8%) were noted to have a FSIQ score above 110, considered to be above average.

Previous studies have reported a high correlation for IQ values in affected siblings with the same deletion mutation, but a poor correlation between unrelated affected individuals with the same deletion. There were 6 pairs of affected brothers, and 7 different mutations shared by 2 or more unrelated individuals in the SCH DMD IQ study group. There was a high correlation between brothers for FSIQ (*r* = 0.98, 95%CI 0.79–0.99, *p* = 0.0008) consistent with previous studies. In contrast with the published literature that unrelated boys with the same mutation were often discordant with respect to intellectual ability [26], [38] the SCH study group data showed that unrelated subjects sharing a *DMD* mutation had a high level of correlation for FSIQ (*r* = 0.83, 95%CI = 0.49–0.95, *p* = 0.0008). The FSIQ correlation of 0.98 for DMD siblings and 0.83 for unrelated DMD-affected individuals with the same mutation is much higher than that previously reported for unaffected male siblings reared together (median correlation of 12 studies 0.38). Rather, it is comparable to the IQ correlation of male monozygotic twins raised together (0.86) [19].

### Association of Mutational Class and Mutation Size with Cognitive Impairment

To determine if the class of mutational event in the *DMD* gene (deletion, duplication, or small point mutation) was correlated with FSIQ, the study group was categorised by mutation class and the mean FSIQs compared by 1-way ANOVA. No significant difference was observed in the mean FSIQ for the different mutational classes (data not shown). To determine if the size of genomic DNA involved in the deletion mutations was correlated with FSIQ, the distribution of FSIQ with respect to both the number of exons and the kilobase pairs of genomic DNA involved in the mutation was compared by a Pearson correlation coefficients but again no significant correlations were identified (data not shown).

### Association of Mutation Location with Cognitive Impairment

#### Proximal versus distal mutations

There is published evidence for a differential effect of proximal versus distal *DMD* mutations on cognitive functioning [1], [25]–[26]. The boundary between proximal and distal groups has been variously set at exon 30 [1] or at exon 45 [25], [26]. In order to determine if correlations could be also be identified in the SCH study group using these criteria the 62 case group was classified into two subgroups based on whether the most 3′ exon involved in the *DMD* gene mutation involved proximal exons only (exons 1–30, or exons 1–45) or whether they also involved the distal exons (exons 31–79, or exons 46–79). For patients with mutations proximal to exon 30 the mean FSIQ was 93 and for those proximal to exon 45 the FSIQ was 91.3. For mutations that were located between exons 31–79 the mean FSIQ was 76.7, and those between exons 46–79 distal mutations the mean FSIQ was 74. The p values for these associations were p = 0.002 and p = 0.0007 respectively using a one-tailed ANOVA for independent samples (Table 2).

**Table 2:** 

Model	Model based on	Proximal group	Intermediate isoform groups (where applicable)			Distal group	F	P	Significance
Model 1 [1]	2 structural groups	Exons 1–30				Exons 31–79	10.44	0.002	*
Model 2 [25], [26]	2 structural groups	Exons 1–45				Exons 46–79	12.82	0.0007	*
Model 3	2 isoform groups	427				260, 140utr, 140pc, 116, 71	7.91	0.0068	*ns*
Model 4	2 isoform groups	427, 260				140utr, 140pc, 116, 71	6.23	0.0155	*ns*
Model 5	2 isoform groups	427, 260, 140utr				140pc, 116, 71	13.72	0.0005	*
Model 6	2 isoform groups	427, 260, 140utr, 140pc				116, 71	11.13	0.0015	*
Model 7	2 isoform groups	427, 260, 140utr, 140pc, 116				71	7.66	0.0076	*ns*
Model 8	3 isoform groups	427		260, 140utr, 140pc, 116		71	7.43	0.0014	*
Model 9	3 isoform groups	427, 260		140utr, 140pc, 116		71	6.39	0.0032	*ns*
Model 10	3 isoform groups	427		260, 140utr, 140pc		116, 71	8.01	0.0009	*
Model 11	3 isoform groups	427, 260		140utr, 140pc		116, 71	6.98	0.002	*
Model 12	3 isoform groups	427, 260, 140utr		140pc, 116		71	9.18	0.0002	**
Model 13	3 isoform groups	427, 260, 140utr		140pc		116, 71	8.43	0.0006	*
Model 14	5 isoform groups	427	260, 140utr	140pc	116	71	5.16	0.0014	*
Model 15	5 isoform groups	427	260	140utr,140pc	116	71	4.48	0.0034	*ns*

Significance threshold 0.003. ns = not significant, * = 1–10-fold and **>10-fold less than the significance threshold.

#### Risk of cognitive disability based of mutation location within Dp140

The foetal brain expressed Dp140 transcript is atypical in that it has a long 5′untranslated region (5′UTR) of 1,041 base pairs (Figure 1). Its promoter lies within intron 44 and first coding exon is exon 51. Subjects with mutations located in the 6½ exons encoding the 5′UTR of the Dp140 isoform (exons 45–50 together with the portion of exon 51 that lies 5′ of c.7381) could therefore be viewed either as having a protein truncating mutation of isoforms Dp427 and Dp260 in which exons 45 to 51 are protein coding exons, or alternatively as a mutation within the 5′UTR of Dp140. As the effect of deletions and point mutations limited to the 5′UTR of Dp140 could not be confidently predicted, but protein truncating mutations in DMD coding exons are of known pathogenicity, subjects with mutations involving the Dp140 transcript were subclassified into two groups. The first group contained those mutations restricted to the 6½ exons within Dp140utr which are interpreted principally as mutations of Dp427 and Dp260 (N = 14, listed as ABB* in Table 1). The second group contained those mutations involving Dp140pc (N = 11, listed as ABC in Table 1), which have been previously shown to have a deleterious effect on the expression of the protein encoded by Dp140 [30], [31]. In 4 instances involving duplication mutations adjacent to exon 45 it was not possible to unequivocally determine whether the Dp140 promoter had been included in the duplication and these samples were excluded from this analysis. A significant difference in mean FSIQ between the 25 subjects in the two groups was identified (p = 0.04, one-tail unpaired t-test), suggestive that mutations in the promoter and coding regions of Dp140 have a more profound effect on levels of protein expression than those in the Dp140 5′UTR.

#### Cumulative effect on FSIQ of mutations in successive dystrophin isoforms

**Figure 2:**
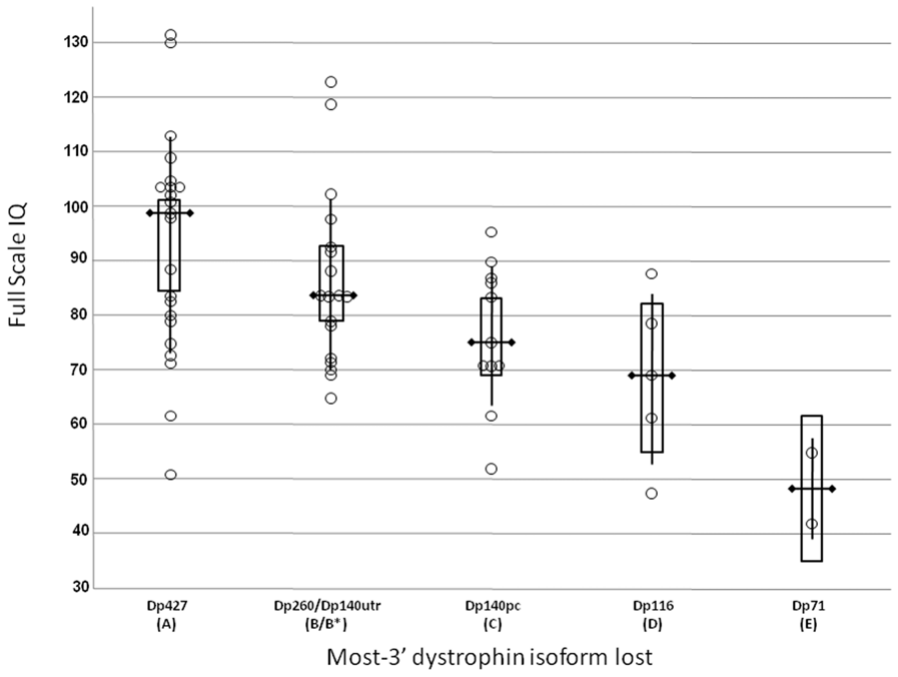
Effect of cumulative loss of dystrophin isoforms on FSIQ. A boxplot representation of patient FSIQ data classified by the most 3′ dystrophin isoform affected by a mutation. Open circles = patient data points; Vertical lines represent ±1 standard deviation of the mean; boxes = 95% confidence intervals of the mean; horizontal bar = median.

Given existing information regarding mutation position and risk of cognitive disability the subset of 58 DMD cases was re-analysed with Dp140utr cases reclassified as primary affecting Dp427 and Dp260 isoforms (Table 2 model 14, and Figure 2). A significant effect of grouped isoform involvement on FSIQ was identified when the data were categorised into 5 groups; Dp427, Dp260+Dp140utr, Dp140pc, Dp116 and Dp71 (df = 4, F = 5.16, p = 0.0014), a model which accounted for a greater proportion of the variance than an alternative 5 group model consisting of Dp427, Dp260, Dp140utr+Dp140pc, Dp116 and Dp71 (model 15; df = 4, F = 4.48, p = 0.0034).

A number of other potential models of were also explored using these data (Table 2). The model that accounted for the greatest proportion of the variance (F = 13.73, p = 0.0005) was model 5, a 2 group model of consisting of those mutations affecting Dp427+Dp260+Dp140utr (mean FSIQ 90, S.E. = 2.9) versus those affecting Dp140pc+Dp116+Dp71 (mean FSIQ 70.4, S.E. = 3.7). The frequency of FSIQ<100 in the two groups was 67% versus 100%; with the comparable frequency of FSIQ of <70 being 10% versus 39%. Model 5 explained a greater percentage of the variance in FSIQ than the other alternative two group models based on assignment of mutations to *DMD* isoforms, and also accounted for more of the variance than 2 group models based on the physical location of mutations proximal of exon 30 (F = 10.44; p =  0.002) or exon 45 (F = 12.82; p = 0.0007). Several three isoform groups were also assessed, but in no instance were the mean FSIQs significantly different in all three groups, as assessed by the Tukey HSD test. It was of interest to note however that the models which clustered mutations in Dp140utr together with Dp260 & Dp427 and apart from Dp140pc (models 5, 12 and 13) explained greater percentage of the variance (average F = 10.4), than those that did not (average F = 7.2).

These data demonstrate that the assignment of mutations to isoform groups was able to explain greater percentages of the variance in FSIQ than consideration of the effects of mutations purely by location, and underscores the importance of the functional consequences of mutation location as underlying some of the liability of cognitive disability in DMD.

## Discussion

This is among the first reported studies of the effect of mutation location on the cognitive capacities of a group of DMD patients for which all mutational classes are represented and where all subjects have been directly assessed using standard intellectual assessment tools. The descriptive IQ results for the SCH study group are representative of previously published studies of cognitive deficits in DMD, with the mean FSIQ, VIQ and PIQ scores being found to be approximately one standard deviation (15 IQ points) below the normative mean of 100, with a VIQ-PIQ deficit of approximately 7 IQ points. These results indicate that the SCH DMD cohort data are likely to be comparable with other DMD cohorts of similar size.

The novel features of this study are that we confirm that the site of the mutation in the *DMD* gene is an important determinant of the risk of cognitive deficit. We also demonstrate here that classification of the risk of cognitive disability based on structural features (deletions prior to or after a specific exon) does not explain as much of the variance in FSIQ in the SCH cohort as does a classification system based on groups of dystrophin transcript isoforms affected by the mutation.

Dystrophin gene expression in the central nervous system is complex and characterised by alternate DMD mRNA transcripts produced from different promoters together with tissue-specific alternative mRNA splicing produces a complex pattern of dystrophin gene related protein expression [39]. The developmental stage, distribution and functions of *DMD* gene products in the CNS, although not well characterised, are believed to be different for each isoform. Differences in the neuropsychological profiles observed among DMD patients have been postulated to be due to the number and type of CNS-expressed isoforms affected. The site of a *DMD* mutation was found to be clearly related to the extent of cognitive deficit. The model which best accounted for this was that where mutations affecting exons 45 to 50 (the 6½ exons comprising the noncoding 5′UTR of Dp140) were considered principally as coding exon mutations whose effect is restricted to Dp260 and Dp427. When categorised in this manner there was a significant difference in the degree of cognitive disability when the mutations that affect the coding regions of the CNS expressed isoforms Dp140pc and Dp71 are clustered together. The findings that groups of *DMD* isoforms explains a greater percentage of the variance in FSIQ needs to seen in the perspective that the magnitude of that effect is still only of the order of 13%.

In 2009 Desguerre and colleagues published a long term follow-up study of a similarly sized cohort of French DMD patients who had not received steroid treatment [1]. In that study additional data relevant to cognitive functioning were gathered over many years of follow-up and when combined explained a larger part of the variance. Of particular interest was their observation that mutation location contributes to cognitive disability but not to motor disability, consistent with the view that that tissue specific effects are mediated at least in part by the effects of *DMD* mutations on the expression of tissue specific isoform expression. A recent paper by Daoud and colleagues [33] investigated the role of mutations affecting Dp140 in DMD, and concluded that mild mental retardation is significantly more frequent with mutations affecting Dp140. Their analysis included a single classification for all mutations in the Dp140 transcription unit and is therefore similar to models 11 and 15 reported in this study.

Although the numbers of patients with Dp71 mutations in this study are small (*N* = 2), the results for these individuals are typical of those in the literature where the vast majority of patients with mutations affecting Dp71 are intellectually disabled [33]. Dp71 is the major product of DMD in the brain but the function of this, the shortest dystrophin isoform remains unknown. Two alternatively spliced isoforms of Dp71, both missing exons 71 and 78 and one also missing exons 72–74 have been identified in human foetal neural tissue and both are regulated during human neural development [40]. Dp71 is also associated with glial end feet and on the basis of this observation Haenggi, *et al* suggested that it may contribute to the proper functioning of the cerebrospinal fluid and blood brain barrier [41]. High level expression of Dp71 has been noted in neonatal and adult brain, particularly in the hippocampus and in some layers of the cerebral cortex [42]. Dp71 expression gradually increases from the embryo stage until adult. Subcellular distribution analysis indicates that Dp71 is mainly recovered in synaptic membranes, microsomes and to a lesser extent in synaptic vesicles and mitochondria [43]. Mice deficient for Dp71 have reduced levels of dystrophin associated proteins in their brain suggesting that Dp71 has a role in the formation and/or stabilisation of the dystrophin associated protein complex in the brain [44].

We also observed a correlation of FSIQ results between related individuals and also for unrelated patients with the same dystrophin mutation suggesting that the reduction in FSIQ observed in boys with DMD has a genetic basis as opposed to environmental factors. The robustness of this observation is diminished by the small numbers in the two groups, reflected in the wide 95% confidence intervals, indicating that this result needs to be validated by further research.

In summary these data represent one of the largest studies of FSIQ and mutational data in DMD patients. The correlation of FSIQ results with the location of the *DMD* mutation is highly suggestive that the risk of cognitive deficit is a result of the cumulative loss of CNS expressed dystrophin isoforms. Further we have presented data which suggest that mutations affecting the Dp140 isoform which are located in the 5′UTR have a lesser effect on FSIQ as compared to those affecting the Dp140 promoter or protein-coding regions.

## Materials and Methods

The study protocol was approved prospectively by the SCH Research Ethics Committee (approval number 04/092QA). Informed consent for neuropsychometric and mutation testing was not requested as these are standard items of clinical care provided to DMD patients in the Sydney Neuromuscular Centre based on international benchmark standards of clinical practice. Data analysis was performed on de-identified patient data in a blinded fashion. Sixty-two boys with Duchenne muscular dystrophy were identified from within this group who met the inclusion criteria for neuropsychological testing and had a *DMD* gene mutation. (The mutation detection rate for DMD patients from this institution is 97% indicating that there is negligible bias in patient selection based on the availability of mutation data). Of these 62 boys, dystrophin isoform assignments could be made for 58 and comprehensive IQ data profiles were available for 53 of these 58. For the remaining 5 subjects partial data profiles were available. The comprehensively studied subjects included six pairs of brothers with the same mutation, and 9 mutations shared by 2 or more unrelated individuals. The mean age at the time of assessment was 9.7 (SD = 2.9, range = 4.25 to 14.83) years.

### Intellectual Assessment

Intelligence testing was performed and the data analysed by a registered clinical psychologist (GAB). Subjects were examined using the Wechsler Intelligence Scales. This consisted of either the Wechsler Preschool and Primary Scale of Intelligence [WPPSI-R] or Wechsler Intelligence Scale for Children [WISC-III], depending on the age at assessment. Children up to the age of 6 years were assessed using the WPPSI-R whereas children over 6 years of age were assessed by the WISC-III.

### Mutation Analysis

Dystrophin gene mutation analysis was performed as part of the study of the SCH B/DMD cohort as previously described [45]. Mutations are described using HGVS nomenclature and the positions of mutations and predicted pathogenic events are reported with respect to reference sequence NM_004006.2 for the Dp427m isoform. Mutations were checked by reference to the Leiden Muscular Dystrophy pages DMD gene reading frame-checker (http://www.dmd.nl/) and analysis with the online sequence variant checking software Mutalyzer (http://eu.liacs.nl/mutalyzer/). The base pair location of the novel Premature Termination Codon (PTC) was determined for out of frame, nonsense and frame-shifting point mutations as the final base of the codon using the output from Mutalyzer and confirmed by visual inspection of the dystrophin gene sequence in Alamut 1.5 (Interactive Biosoftware). For in-frame mutations pathogenicity was inferred from published data of the effects of the mutation on dystrophin protein functional domains.

Dystrophin mutations were assigned to mRNA isoforms as follows (Figure 1). Mutations were used to determine the position of PTCs as outlined above. Mutations were assigned to isoform Dp427m (‘A’) if their PTCs were located 5′ of the first base of the ATG of the Dp260 isoform at c.4072-296. Mutations were deemed to affect both Dp427 and Dp260 (‘AB’) if the PTC lay between ATG of Dp260 at c.4072-296 and exon 1 of Dp140 at c.6438+61447. Mutations were deemed to affect Dp427, Dp260 and Dp140utr (‘ABB*’) if their PTCs were located between the noncoding exon 1 of Dp140 at c.6438+61447 and 5′ of the ATG of Dp140 at c.7381 *and* left the Dp140 promoter intact. Mutations that removed the Dp140 promoter and had a PTC located in the coding region of Dp140 between the initiator ATG at c.7381 and that of Dp116 at c.8218-790 were deemed to affect isoforms Dp427m, Dp260 and Dp140 (‘ABC’). Four isoforms Dp427, Dp260, Dp140 and Dp116 (‘ABCD’) were deemed to be involved if a PTC was located between the initiator ATGs of Dp116 at c.8218-790 and Dp71 at c.9225-5813. All 5 isoforms (‘ABCDE’) were deemed involved if a PTC was located distal to the ATG of Dp71 at c.9225-5813.

For the Dp140 isoform the presence or absence of its unique first coding exon and adjacent 5′ promoter region was determined in all patients with a deletion mutation where the last deleted exon was exon 44, or the first deleted exon was exon 45. The integrity of the Dp140 unique first exon was established as described in Lidov *et al.* [46]. The integrity of the Dp140 promoter was determined by amplification of microsatellite IVS44SK12, as described by Kochling *et al.* [47]. Dystrophin exon 8 was co-amplified as a control, but as this technique is not quantitative the results from 4 subjects (1× exon 45 duplication and 3× exon 45–52 duplications) were not included in all parts of the analysis.

### Statistical Analyses

For the analysis of the cognitive profile of the FSIQ study group, statistical analyses were performed using one sample *t*-test compared to normative Wechsler Scale mean values and Pearson correlation coefficient for patients having the same dystrophin mutation. The likelihood of a normal distribution of values was assessed using the D'Agostino & Pearson omnibus normality test. In each case *p*<0.05 was considered significant. The Pearson correlation coefficient was calculated to determine correlations between FSIQ results for DMD sib-pairs and unrelated pairs with the same dystrophin mutation and also for correlations between FSIQ and the number of exons deleted in deletional cases of DMD. The paired Student's *t*-test was used to determine the similarity of the differences in paired FSIQ data for sib-pairs compared to unrelated–pairs with the same dystrophin mutation. One way ANOVA was used to assess differences between the 3 major mutational classes associated with DMD and FSIQ. For the hypothesis that the loss of groups of more distal isoforms cumulatively affected FSIQ a one way ANOVA was performed on the classification methods and amount of the variance accounted for by each model was quantified using an F statistic. A Bonferroni correction for significance was applied to take into account multiple ANOVA tests performed and the adjusted threshold for significance set at 0.05 (prior significance)/15 (the number of models tested) = 0.003.
